# Preclinical Evaluation of Long-Term Neuroprotective Effects of BDNF-Engineered Mesenchymal Stromal Cells as Intravitreal Therapy for Chronic Retinal Degeneration in Rd6 Mutant Mice

**DOI:** 10.3390/ijms20030777

**Published:** 2019-02-12

**Authors:** Renata Lejkowska, Miłosz Piotr Kawa, Ewa Pius-Sadowska, Dorota Rogińska, Karolina Łuczkowska, Bogusław Machaliński, Anna Machalińska

**Affiliations:** 1 Department of General Pathology, Pomeranian Medical University in Szczecin, 70-111 Szczecin, Poland; renatalejkowska@gmail.com (R.L.); kawamilosz@gmail.com (M.P.K.); ewapius@wp.pl (E.P.-S.); doroginska@gmail.com (D.R.); karolinaluczkowska58@gmail.com (K.Ł.); 2 First Department of Ophthalmology, Pomeranian Medical University in Szczecin, 70-111 Szczecin, Poland

**Keywords:** MSC, BDNF, rd6, retinal degeneration, regenerative medicine, tissue imaging, OCT, lentiviral vectors

## Abstract

This study aimed to investigate whether the transplantation of genetically engineered bone marrow-derived mesenchymal stromal cells (MSCs) to overexpress brain-derived neurotrophic factor (BDNF) could rescue the chronic degenerative process of slow retinal degeneration in the rd6 (retinal degeneration 6) mouse model and sought to identify the potential underlying mechanisms. Rd6 mice were subjected to the intravitreal injection of lentivirally modified MSC-BDNF or unmodified MSC or saline. In vivo morphology, electrophysiological retinal function (ERG), and the expression of apoptosis-related genes, as well as BDNF and its receptor (TrkB), were assessed in retinas collected at 28 days and three months after transplantation. We observed that cells survived for at least three months after transplantation. MSC-BDNF preferentially integrated into the outer retinal layers and considerably rescued damaged retinal cells, as evaluated by ERG and immunofluorescence staining. Additionally, compared with controls, the therapy with MSC-BDNF was associated with the induction of molecular changes related to anti-apoptotic signaling. In conclusion, BDNF overexpression observed in retinas after MSC-BDNF treatment could enhance the neuroprotective properties of transplanted autologous MSCs alone in the chronically degenerated retina. This research provides evidence for the long-term efficacy of genetically-modified MSC and may represent a strategy for treating various forms of degenerative retinopathies in the future.

## 1. Introduction

Inherited or acquired retinal degeneration involving the retinal pigment epithelium (RPE) and photoreceptors is a major cause of loss of vision leading to complete blindness throughout the world. Retinal degeneration involves complex processes and the pathophysiological mechanisms are not fully known. Since the human population is constantly aging, there is an enormous need to develop new treatment approaches [1]. In the past, numerous strategies have been performed to stop the degeneration process in the retina and to replace damaged cells, but their therapeutic effects were very limited [2]. This inefficacy is because the genetic and phenotypic heterogeneity of the retinal degeneration process makes the development of a single therapeutic approach a very challenging process [3]. Several factors that contribute to retinal neurodegeneration have been partially identified, including augmented oxidative stress, and dynamic metabolic changes in retinal cells owing to the activation of inflammatory responses and decreased availability of vital trophic factors.

Neurotrophins (NTs) are a large family of different growth factors, including main members such as the neural growth factor (NGF), the brain-derived neurotrophic factor (BDNF), neurotrophin-3 (NT-3), and neurotrophin-4/5 (NT-4/5) [4]. The major role of NTs is the promotion of neuronal survival, which is mediated through the interaction of NTs with receptor tyrosine kinases (TrkA, TrkB, and TrkC) [5]. Stimulation of the TrkB receptor, which is a target for both NT-4 and BDNF, results in activation of several intracellular signaling cascades that are responsible for cell survival and proliferation together with cell migration, differentiation and neural tissue development [4]. Some of the Trk-dependent cascades are also responsible for preventing apoptosis [6]. It was found that NTs and their receptors are also locally produced in the retina and BDNF was detected in Müller cells [7], retinal ganglion cells and cells of the inner nuclear layer [8]. It is postulated that several neurotrophic factors may contribute to retinal cell survival and development and cell neuroprotection in case of injury to the retina. Exogenously applied BDNF was reported to increase the number of neuronal progenies [9]. In the retina, an intravitreal supplementation of exogenous BDNF enhanced the survival of injured adult retinal cells [10,11], however this effect was only temporary and was unsuccessful in the long-lasting prevention of retinal cell death [12].

Several studies have reported that neuroprotective effects could be supported by adult stem cell transplantation to the site of the damaged retina [13,14]. Mesenchymal stromal cells (MSCs) are one of the most commonly tested cells and have been proven effective in preclinical studies of many disorders, including neurodegenerative diseases [15]. In certain animal models of retinal degeneration, MSCs therapies showed neuroprotective effects [16]. They have the capability to regulate the promotion of photoreceptor survival by their anti-inflammatory, anti-apoptotic, immunomodulatory, and angiogenic effects, which are based on their secretion of cytokines [17] and growth factors [18]. The most common sources of MSCs are bone marrow and fat tissue [19], which provide an abundant source of those cells in the clinical setting. Therefore, the genetic modification of MSC, permitting the production of selected proteins, can provide cellular products of higher quality for therapeutic use, due to the more stable expression profile and uniform secretion of the desired biologically active substances. During the past decade several strategies for introducing growth factor genes into MSCs have been developed, among which the lentiviral vectors transduction indicates much greater efficacy compared to other vectors routinely used in gene therapy and MSCs modifications [20]. Regarding this notion, a lentivirus-based engineering of MSCs for the constant production of neurotrophins has been proposed as a promising method for the long-term delivery of neuroprotective substances to the damaged retinas in various preclinical studies [15]. Likewise, our group previously found that NT-4-engineered MSCs actively produced exogenous neurotrophin-4 after cell transplantation to the acutely-injured retinas in vivo and significantly rescued the damaged retinal cells [21]. Moreover, the successful transplantation of genetically engineered MSCs secreting BDNF in the central nervous system (CNS) was already performed in axotomized retina and in laser induced injury [22,23]; however, to our knowledge, there are no studies that have specifically analyzed the effect of the continuous delivery of BDNF via combined cellular and gene-based therapy in a model of chronic retinal degeneration. To investigate whether there is a rescue potential of MSC-BDNF in inherited retinal degeneration, we used a well-established animal model of slow retinal degeneration—rd6 mutant mice. The rd6 mouse is a biologic model of retinal pigment epithelium-based autosomal recessive retinitis pigmentosa caused by the mutation in the MFRP (membrane-type frizzled related protein) gene. In these animals, the retina has a normal cell composition at birth, although during the postnatal life there is a continuous degeneration of photoreceptors. Significant decrease in thickness of outer nuclear layer is reported in four- and five-month old individuals, and even more by those aged seven months, especially due to the reduction of the photoreceptor cell number. Near complete loss of vision (1 layer) is noted at the age of 24 months [24,25]. Therefore, we chose this model to characterize the molecular mechanisms underlying the long-term protective effect of transplant therapy with MSC-BDNF and compare it with the effect of MSCs alone.

## 2. Results

### 2.1. Evaluation of Ex Vivo Genetic Modification of MSCs for Effective BDNF Production

**Figure 1:**
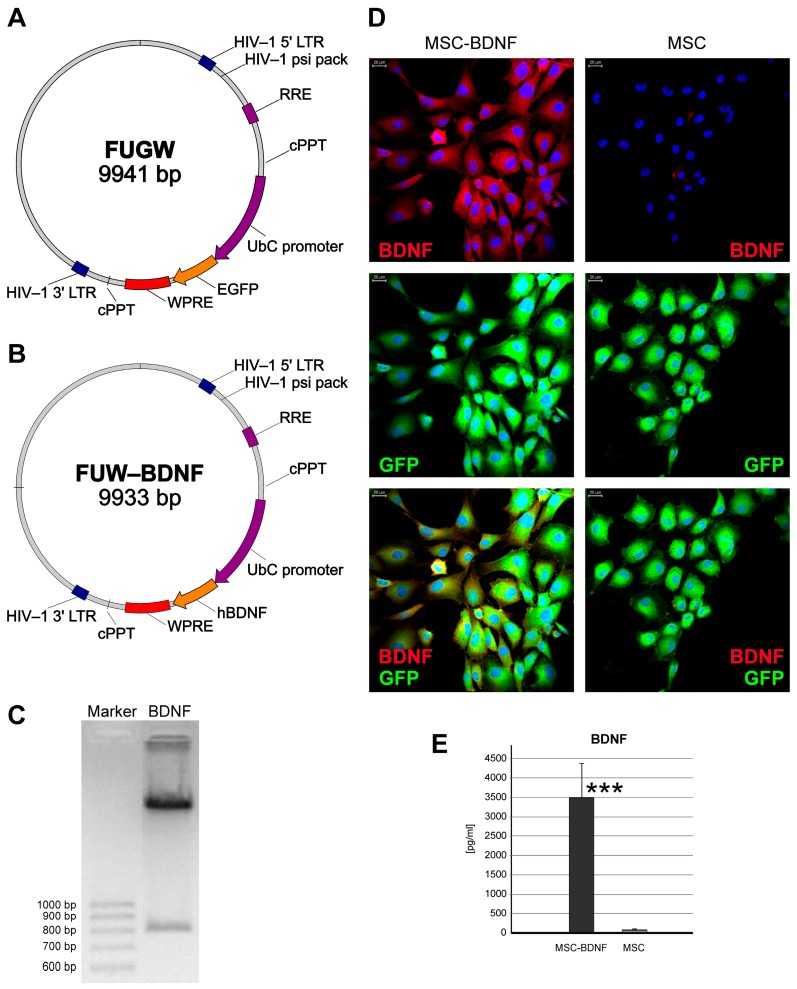
Characterization of lentiviral MSCs transduction efficiency. The schemes of plasmids used for lentivirus production for subsequent murine MSCs transduction are shown. The lentiviral backbone plasmid (FUGW) contained the green fluorescent protein (GFP) coding sequence (**A**) that was removed to insert the human BDNF sequence and then FUGW-BDNF plasmid was created (**B**) for relevant lentiviral vectors production. The correct band for BDNF insert (765 bp) was observed under ultraviolet (UV) light in agarose gel (**C**). Quantitative analysis of BDNF levels from MSC-BDNF and unmodified MSC cultures in vitro (**D**). Noninfected control MSCs produced only trace amount of BDNF, whereas production of BDNF in MSC-BDNF culture was approximately 35-fold increased. These data were corroborated by double immunofluorescent staining of BDNF and GFP proteins for their qualitative expression and co-expression analysis (**E**). Scale bar: 20 µm, *** *p* < 0.001.

In our study we used two types of MSC—sunmodified MSCs and transduced ones, both expressing green fluorescence. To infect the collected murine MSCs, the recombinant lentivirus with expression of human BDNF (Lv-UbC-BDNF) was used. Both MSC forms used in this experiment presented a typical MSC phenotype, according to their morphology (i.e., spindle-like shape and plastic adherence) as shown in Figure 1D, and the expression of Sca-1, CD90 and CD105 antigens, with no CD31 expression, as detected by flow cytometry (results were approximately the same as shown in Reference [21]). Moreover, following the infection with the BDNF-encoding lentivirual vectors, the vast majority of cultured MSCs were positive for BDNF protein (Figure 1D). Finally, ELISA results revealed a significant increase in the BDNF concentration (over 35-fold increase, *p* < 0.0001) in medium collected from the BDNF–positive MSC culture compared to the uninfected MSC in the same conditions (Figure 1E).

### 2.2. Homing, Migration, and Survival of Transplanted MSC within Injured Retina

**Figure 2:**
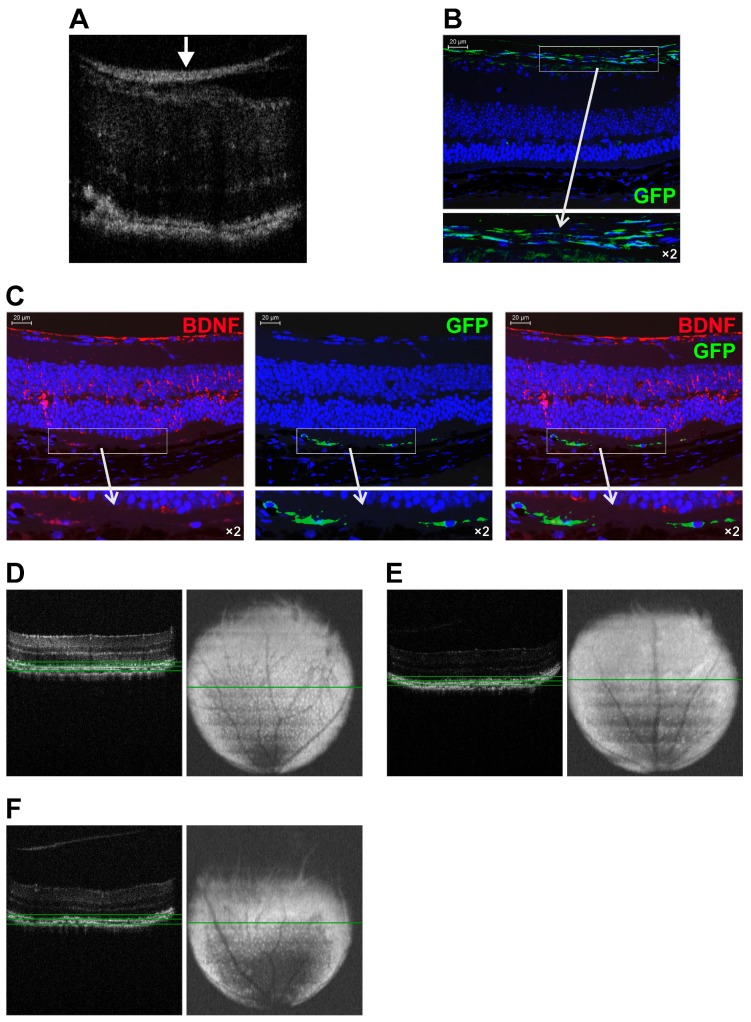
Long-term follow-up of genetically modified MSC-BDNF and MSC trafficking and homing at different time points post-intravitreal transplantation in rd6 mice. A representative SD-OCT image of chronically degenerated retina of rd6 mouse at the 28th day after intravitreal MSC-BDNF injection (**A**). A hyperreflective streak of the accumulated MSC (white arrow) at the vitreoretinal interface is observed. A representative fluorescence image of degenerated retina of rd6 mouse at 28 days after intravitreal MSC injection (**B**). At this time point, the vast majority of the injected GFP-positive cells (green) were found to be located at the vitreoretinal interface and in the superficial ganglion cell layer. A representative fluorescence images of degenerated retina of rd6 mouse at three months after intravitreal MSC-BDNF injection (**C**). At this time of the experiment, the injected GFP-positive cells (green) were found to be aligned along the RPE-photoreceptor junction and showed double immunostaining against BDNF (red). A representative retinal volume intensity projections of OCT scans of rd6 control mouse (**D**), after intravitreal MSC-BDNF injection (**E**) and MSC alone transplantation (**F**) at the third month of the experiment. At this time of the experiment, the considerable reduction of the retinal white spots that correspond to macrophages and monocytes at the level of retinal pigment epithelium was observed only in eyes after intravitreal MSC-BDNF injection. Green lines indicate the retinal level where the volume intensity projection image (VIP) was captured. Scale bar: 20 µm.

First, we wondered whether any differences in the homing mechanisms between infected and uninfected GFP positive MSCs exist and if they could be efficiently delivered to the retina of rd6 mice using intravitreal pars plana injection. The main goal was to assess the MSCs ability to traffic from the vitreous body to damaged retina and their final homing in retina. Thus, we monitored the eyes on the 28th day and at three months after transplantation of the cells using the spectral domain optical coherence tomography (SD-OCT) technique. After MSC-BDNF transplantation, the OCT B-scans showed hyperreflective streaks at the vitreoretinal interface (Figure 2A), which were detectable throughout the entire experimental period. Importantly, the intensity of that bright streak representing the injected MSC cells decreased during the time course of the experiment in the case of MSC-BDNF but not in MSC alone. This might indicate that a strong overexpression of BDNF stimulates the effective migration of transplanted MSC-BDNF from the vitreous body toward the degenerated retinal tissue in rd6 mice, whereas unmodified MSCs are not able to migrate towards the deep retinal layers and remain in the vitreoretinal interface.

To confirm this observation and to better define the localization of transplanted MSCs, we analyzed the histologic specimens of retinas from rd6 mice after transplantation using immunofluorescence technique. We applied the endogenously expressed fluorescent GFP protein as a marker to evaluate the migration, location, and long-term survival of MSC after their grafting. We observed that on the 28th day, the transplanted MSC-GFP cells were still located in the posterior eyecup at the vitreoretinal interface and in the superficial ganglion cell layer (Figure 2B). In contrast, in the course of the experiment, MSC-BDNF migrated toward different retinal layers and were mostly found in the RPE layer and among photoreceptors (Figure 2C), where they were able to survive for up to three months in a good metabolic state expressing marker GFP. Furthermore, the immunofluorescence analysis of the retinas after the intravitreal administration of MSC-BDNF showed double-positive cells stained for BDNF and GFP together, indicating the localization of MSC-BDNF cells along the RPE-photoreceptor junction on the third month of the experiment (Figure 2C). These results may suggest that MSC after intravitreal transplantation preferentially migrates toward the deep retinal layers selectively damaged in the rd6 mouse model, and they could maintain their biological activity of BDNF production in vivo for a longer period of time, up to several months.

Essentially, the volume intensity projections of OCT scans of chronically degenerated retinas in rd6 mice revealed an abundant number of distinct subretinal white spots oriented in a regular pattern across the retina that correspond to macrophages and monocytes typically juxtaposed to the retinal pigment epithelium (Figure 2D). In contrast, we observed that on the third month after intravitreal MSC-BDNF injection, there was a notably decreased quantity of such white visible spots detected in the volume intensity projections of OCT scans (Figure 2E). These results may account for the corresponding local macrophage depletion at the site of retinal degeneration after MSC-BDNF transplantation. This may indicate a reduction in proinflammatory and scavenging functions related to favorable local activity of transplanted MSC-BDNF, which in turn decrease a stimulus for macrophage and other inflammatory effector cells infiltration.

### 2.3. Long-Lasting BDNF Secretion by Engineered MSC and Its Biological Effects

**Figure 3:**
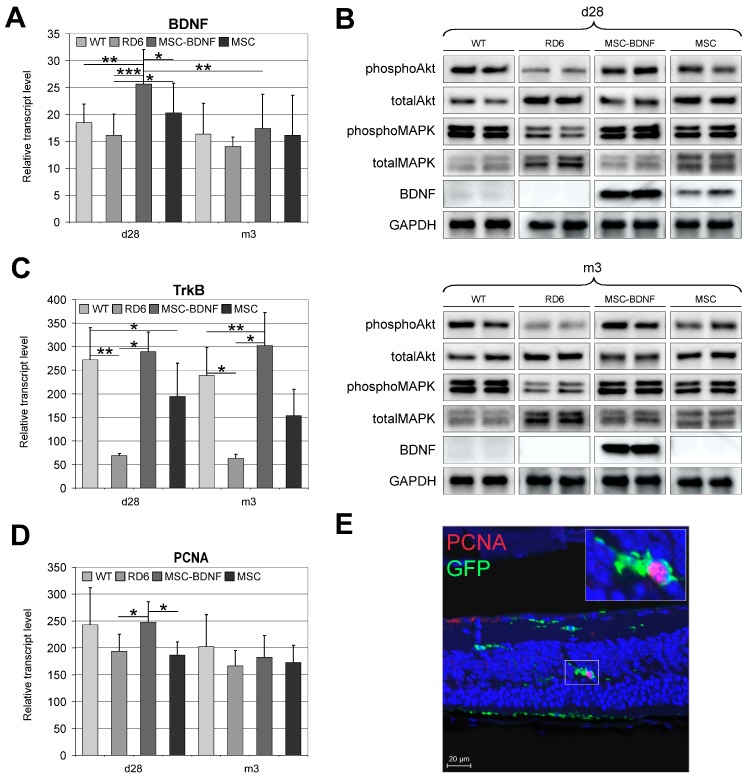
Long-term follow-up of BDNF production and its biological function at different time points post-intravitreal MSC-BDNF transplantation. BDNF mRNA (**A**), BDNF, phosphoAkt, totalAkt, phosphoMAPK and totalMAPK protein (**B**) expression was detected in retinas from eyes treated with MSC-BDNF, and their levels were significantly increased post-transplantation compared to other groups: at 28 days in case of mRNA and protein and at three months in the case of BDNF protein only. We also observed increased TrkB gene expression in retinas at 28 days and three months after MSC-BDNF and MSC alone transplantation compared to rd6 and wild type (WT) (**C**). The follow-up of retinal cell proliferation at different time points post-intravitreal transplantation in rd6 mice was also performed. Quantitative analysis of proliferating cell nuclear antigen (PCNA) mRNA expression revealed that their levels were significantly increased in retinas from eyes treated with MSC-BDNF at 28 days post transplantation compared with those in eyes treated with the PBS and MSCs alone (**D**). Double-stained sections for PCNA and GFP (endogenous marker of transplanted MSC) used to visualize and localize proliferating cells revealed the extraordinary PCNA protein concentration in MSC-BDNF transplanted 28 days previously (**E**). Representative images of the performed analyses are shown. Scale bar: 20 µm. Reference gene used for qRT-PCR analysis was glyceraldehyde 3-phospate dehydrogenase (GAPDH). Mean values ± SD are presented in the diagrams, * *p* < 0.05, ** *p* < 0.01, *** *p* < 0.001 (*n* = 7/group/time point).

On the 28th day and three months post-MSC administration, the expression of BDNF was analyzed in the retinas collected from the following groups of rd6 mice: (1) With transplantation of MSC-BDNF alone, (2) with transplantation of MSC alone (unmodified cells), and (3) with injection of phosphate buffered saline (PBS) alone (control for the cell administration procedure). Four weeks after unmodified MSCs transplantation, the retinal expression of BDNF was low, both at the mRNA (Figure 3A) and protein (Figure 3B) level. In contrast, the retinas transplanted with MSC-BDNF presented a significantly increased expression of mRNA for BDNF compared to those transplanted with other treatments (Figure 3A). Similarly, the BDNF protein expression was strongly augmented in the eyes treated with MSC-BDNF compared with that in eyes injected with MSC alone or with PBS alone (Figure 3B). However, during the third month of the experiment, the mRNA transcripts encoding BDNF decreased significantly compared to day 28 post-transplantation (Figure 3A). The expression of BDNF protein remained at a constant expression level (Figure 3B). These results indicate that genetically modified MSC used to express exogenous BDNF, which were transplanted into slowly degenerating retinas of rd6 mice, may produce the neurotrophic growth factor—BDNF—locally at the protein level for at least three months after their transplantation.

Next, we sought to analyze whether locally produced BDNF may biologically act in the target retinal tissue. Therefore, we examined the expression of the TrkB receptor, which is specific for BDNF, using quantitative reverse transcription polymerase chain reaction (qRT-PCR). Indeed, we observed a significantly increased expression of mRNA encoding TrkB in retinas after MSC-BDNF transplantation both at day 28 and at the third month of the experiment compared to that after the transplantation of MSCs alone or PBS control (Figure 3C). This finding indicates that BDNF could induce its activity in retinal tissue due to the availability of transcripts of its specific receptor and potentially might activate it in the cells.

To more closely examine the BDNF-dependent signaling network, we analyzed the expression of key members of signal transduction pathways related to the TrkB receptor and its ligand, BDNF. Analysis of Western blot membranes demonstrated substantial increases in the expression levels of phosphorylated protein kinase B (Akt) and mitogen-activated protein kinase (MAPK) in the retinas from MSC-BDNF-treated mice, and to a lesser extent in retinas from unmodified MSC, compared with those levels in control rd6 retinas (Figure 3C). These data suggest that BDNF abundantly released from genetically-engineered MSCs is highly bioactive and may induce the persistent activation of Akt and MAPK-related pathways through their phosphorylation during long-term observation and thus contribute to the neuroprotective and survival functions of BDNF-related signaling.

Furthermore, to determine whether the TrkB-dependent signaling pathways are biologically active after MSC-BDNF transplantation, we assessed the proliferative potential in the target tissue after MSC-BDNF transplantation. Therefore, we analyzed the expression of proliferating cell nuclear antigen (PCNA) by determining the level of its mRNA transcripts. We found that at 28 days post transplantation, the PCNA mRNA quantity was significantly increased in the eyes treated with MSC-BDNF compared with those control eyes injected with MSC alone or PBS (Figure 3D). Additionally, the precise localization of the PCNA-positive cells was determined by an immunohistofluorescence analysis of retinas collected from MSC-BDNF-treated mice using the anti-PCNA antibody. We found that PCNA protein was present mainly in the MSCs expressing BDNF, which were migrating across the degenerated retinas of rd6 mice (Figure 3E). The results may suggest the beneficial role of exogenous BDNF expression for cell proliferation, especially through the autocrine mode of its actions.

### 2.4. Induction of Anti-Apoptotic Signaling in Degenerated Retinas during Long-Term Observation

**Figure 4:**
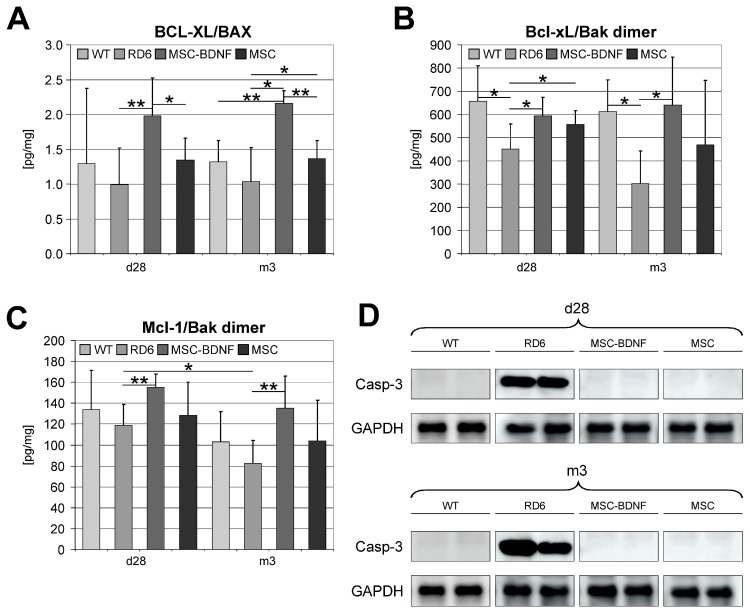
The expression profile of selected apoptosis-related molecules in retinas treated with MSC-BDNF or MSC alone at different time points (at 28 days and three months post transplantation) and compared to WT and rd6 mice. The mRNA expression of Bcl-xL and BAX genes was determined by the quantitative PCR and the relative ratio Bcl-xL/BAX was calculated (**A**). The concentration of Bcl-xL and Bak protein dimer was determined by specific Luminex (**B**) Similarly, the concentration of Mcl-1/Bak dimer protein was measured (**C**). Caspase-3 protein expression was determined by Western blot, which revealed a lack of expression of this form in WT mice and rd6 after cell transplantation compared to rd6 mice with no treatment (**D**). GAPDH served as loading. A representative image is shown. Mean values ± SDs are presented in the diagrams, * *p* < 0.05, ** *p* < 0.01 (*n* = 7/group/time point).

Next, we continued to characterize the biologic activity of long-lasting BDNF secretion by analyzing cardinal cellular processes, such as programmed the cell death signaling and activity. Therefore, to determine whether the TrkB-dependent signal transduction pathway controlling apoptosis is also biologically active after MSC-BDNF transplantation, we analyzed the expression of several important anti- and pro-apoptotic factors in retinas collected from all experimental groups of rd6 mice in this study. Apoptosis is strictly controlled by the balance between pro- and anti-apoptotic members of the Bcl-2 protein family. Therefore, we performed a quantitative molecular analysis of mRNA expression for the important anti-apoptotic gene BCL-XL (B-cell lymphoma-extra-large protein) and the main pro-apoptotic gene BAX (Bcl-2 associated X protein). We observed an increased expression of anti-apoptotic genes in the mRNA from retinas after MSC transplantation compared to that in untreated retinas. Next, we compared the value of the Bcl-xL/BAX mRNA ratio that defines tissue propensity to block apoptosis, and it was significantly higher in MSC-BDNF-treated mice compared with that in mice injected with MSC alone or PBS (Figure 4A). This finding suggests that local exogenous BDNF production and its secretion from MSC-BDNF is strongly involved in modulating the expression of selected genes encoding apoptosis-regulating proteins. To further confirm this study, we analyzed protein lysates from retinas treated with MSC-BDNF and compared them with retinas injected with unmodified MSC or PBS. As shown in Figure 4B,C, we performed immunoassays and observed the significantly increased concentration of the Bcl-xL/Bak (Bcl-2 homologous antagonist killer) and the Mcl-1 (induced myeloid leukemia cell differentiation protein)/Bak protein dimers, respectively, in the MSC-BDNF-treated group compared to that in the other examined groups at both time points analyzed in the experiment. Next, to finally confirm the anti-apoptotic activity of BDNF produced locally in the retina, we used a Western blot analysis to compare the expression of Caspase-3 protein. As shown in Figure 4D, we were not able to detect Caspase-3 in immunoblots of the lysates from retinas treated with MSC-BDNF as well as treated with MSC alone both at day 28 and month 3. In contrast, the Caspase-3 was expressed in degenerated retinas of animals in the control group. Overall, these results indicate that MSC-BDNF transplantation may potentially reduce the activity of apoptosis in degenerated retinas of mature rd6 mutant mice. These findings also suggest that BDNF locally released from MSC-BDNF after their transplantation is biologically active, affecting the crucial cellular processes in the retinal tissue, such as proliferation and apoptosis, for at least three months after the experimental therapy onset.

### 2.5. Neuroprotective Properties of Transplanted Cells after MSC-BDNF Treatment during Long-Term Observation

**Figure 5:**
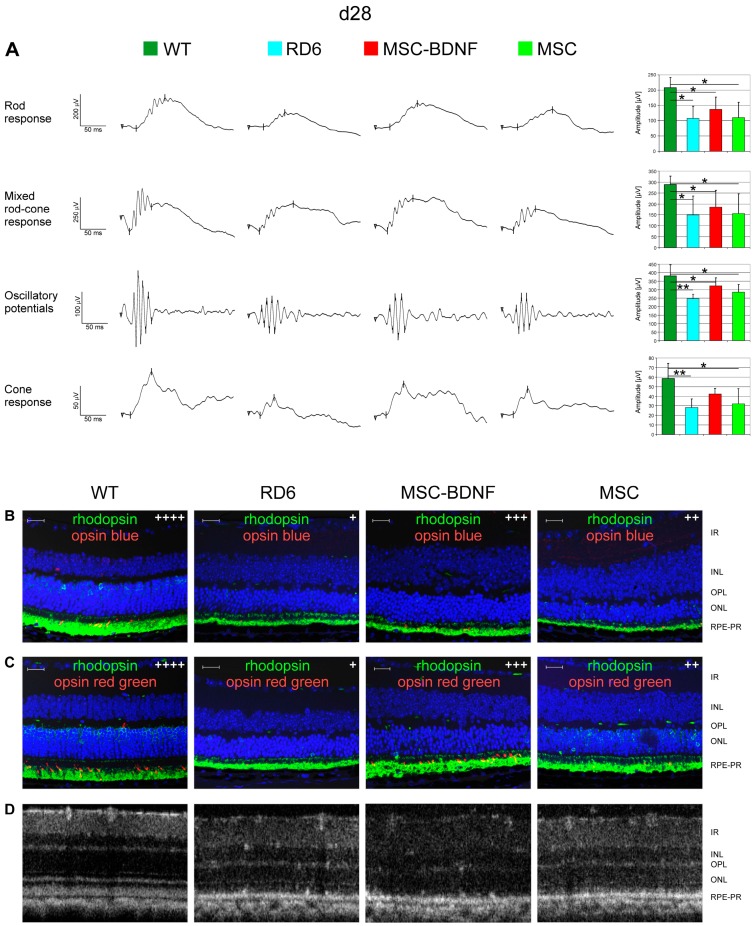
Effects of in vivo experimental therapy with genetically transduced MSC-BDNF and unmodified MSC on the retinal function and OCT-based retinal morphology at 28 days post transplantation compared to rd6 mice with no treatment and WT mice. The representative ERG responses recorded after cell injection are shown (**A**). The b-wave amplitude measurements are presented as the mean ± SD immunohistofluorescence co-staining for rhodopsin (green) with opsin blue (red) in (**B**) and rhodopsin (green) with opsin red/green (red) in (**C**) are displayed in the representative retinal specimens from all the groups with semi quantitative evaluation (signal intensity was marked with subsequent methodology: “+” when less than 5 immunopositive cells present in the image, “++” for 6–15 of visible positive cells, “+++” for 16–30 of positive stained cells, “++++” more than 30 immunopositive cells. The control rd6 mouse retinas (left column) are almost completely negative for both cone opsins, whereas MSC-BDNF-treated retinas displayed regeneration of cones as evidenced by positive immunoreactivity for red/green cone opsin (middle column). The representative OCT images of the retinal sections with their typical layered structure are presented in (**D**). Retinal layers were marked both at histological images and OCT as follows: IR- inner retina, INL- inner nuclear layer, OPL- outer plexiform layer, ONL- outer nuclear layer, RPE-PR–retinal pigment epithelium and photoreceptors. Scale bar: 20 µm. * *p* < 0.05; ** *p* < 0.01.

**Figure 6:**
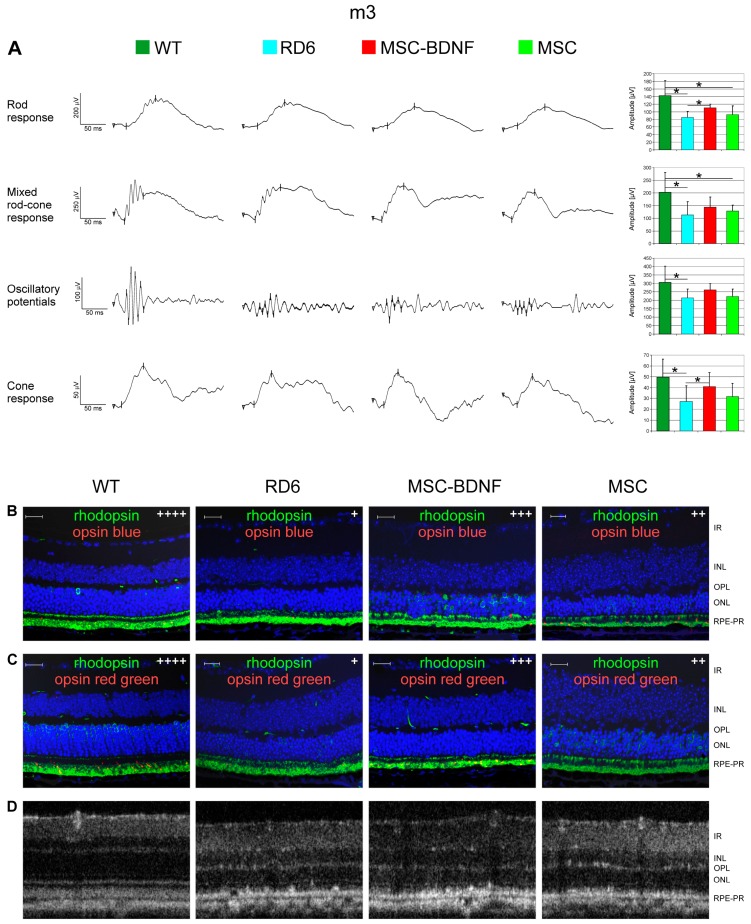
Effects of in vivo experimental therapy with genetically transduced MSC-BDNF and unmodified MSC on the retinal function and OCT-based retinal morphology at three months post transplantation compared to rd6 mice with no treatment and WT mice. The representative ERG responses recorded after cell injection are shown (**A**). The b-wave amplitude measurements are presented as the mean ± SD. Immunohistofluorescence co-staining for rhodopsin (green) with opsin blue (red) in (**B**) and rhodopsin (green) with opsin red/green (red) in (**C**) are displayed in the representative retinal specimens from all the groups with semi quantitative evaluation (signal intensity was marked with subsequent methodology: “+” when less than 5 immunopositive cells present in the image, “++” for 6–15 of visible positive cells, “+++” for 16–30 of positive stained cells, “++++” more than 30 immunopositive cells. The control rd6 mouse retinas (left column) are almost completely negative for both cone opsins, whereas MSC-BDNF-treated retinas displayed a regeneration of cones as evidenced by the positive immunoreactivity for red/green cone opsin (middle column). The representative OCT images of the retinal sections with their typical layered structure are presented in (**D**). Retinal layers were marked both at histological images and OCT as follows: IR- inner retina, INL- inner nuclear layer, OPL- outer plexiform layer, ONL- outer nuclear layer, RPE-PR–retinal pigment epithelium and photoreceptors. Scale bar: 20 µm. * *p* < 0.05.

To assess whether the local production of BDNF exogenously provided to the retinal level in the eye can induce prolonged changes in retinal biology and function, we monitored the bioelectrical retinal response using sensitive ERG equipment at day 28 and month 3 after transplantation. We observed that mean values of b-wave amplitudes both in rod and cone responses were noticeably higher in MSC-BDNF-treated retinas (mostly in the MSC-BDNF-treated group) than in control eyes both at day 28 (Figure 5A) and month 3 (Figure 6A) after injection. To determine the cellular basis of this considerable functional rescue, we analyzed collected retinas immunohistochemically with antibodies specific for rod or cone opsins. The analysis of double immunohistofluorescence staining for rhodopsin with opsin blue or with opsin red/green at day 28 (Figure 5B,C) and month 3 (Figure 6B,C) after transplantation confirmed the ERG recordings. We noticed the increased expression of the tested light-sensitive proteins in retinas after cell administration compared to that after the control, in which the presence of opsins was almost undetectable in our analysis. The increased expression of opsin proteins responsible for both rods and cones responses (but mainly cones) was especially seen in retinas after MSC-BDNF transplantation and continued for at least three months after cell injection.

## 3. Discussion

Retinal degeneration is caused by a series of metabolic disturbances in the retina, which leads to progressive retinal atrophy and eventually to the complete degeneration of photoreceptors causing sight worsening and subsequent blindness. Therefore, searching for innovative effective therapies used in this condition as a vital medical need. There are currently no alternative therapeutic prospects for intractable ocular diseases, other than gene and cellular therapies, hence, various investigations have recently focused on them. The transplantation of different cells is a new option to treat neurodegenerative disorders. Therapeutic cellular strategies are based on the trophic activity of stem and progenitor cells (SPCs) producing various cytokines, including growth factors and extracellular matrix compounds, which regulate the growth, differentiation, and survival of various types of cells [26]. In previous decades, various cell types have been verified and tested for retinal neuroregeneration, including mesenchymal stromal cells, hematopoietic, embryonic and induced pluripotent stem cells, among others [3,27]. Growing evidence has documented that neuro-functional deficits can be reduced by bone marrow-derived cells in experimental animal models of retinal degeneration [28]. Our group observed that intravitreal administration of bone marrow (BM)-derived Lin-negative cells (Lin^−^) cells into murine eyes after acute retinal injury led to the integration of the Lin^−^ cells in outer retinal layers, thereby improving the morphological retinal structure and inducing molecular changes such as the downregulation of pro-apoptotic signaling pathways and the induction of neuro-regeneration, partially through the secretion of neurotrophic factors, especially BDNF [29]. Additionally, transplanted Lin^−^ cells differentiated locally in damaged retina into cells with a macrophage-like phenotype. Our other study determined that the intrathecal application of autologous BM-derived Lin^-^ cells provides strong anti-inflammatory support in the early stage of neuronal degeneration in chronic neurodegenerative disease, such as ALS, which is a disease with a pro-inflammatory background [30]. 

Prominent data indicate mesenchymal stromal cells are a feasible and preferred candidate for cell-based therapy for various neurological disorders [31,32,33]. From this perspective, the most important features of these cells are as follows: (1) Availability from autologous sources independent of a patient’s age; (2) extensive expansion in vitro; (3) immunomodulatory “bystander” function after transplantation in vivo; (4) potential to protect, repair or eventually replace impaired or dysfunctional host cells [34]. Importantly, MSCs fail to trigger an immune response when transplanted to other tissues, as the MSC-derived factors inhibit the proliferation of several types of immunologically competent cells [35]. Thus, the immunosuppressive properties of MSCs permit an immunological acceptance of cells transplanted into the vitreous cavity of the eye. After a transplantation into the eyes of animals with slow retinal degeneration, such as Royal College of Surgeons (RCS) rats or mouse model of retinitis pigmentosa, MSCs sparsely differentiated into cells with glial characteristics, but not to mature photoreceptors or RPE cells [36]. However, they exerted the protective effects on endogenous photoreceptors and RPE cells [37]. The reduction in retinal ganglion cell (RGC) death in animal models of glaucoma [38] and optic nerve transection [39,40] has also previously been demonstrated for BM-derived MSCs. Intravitreally transplanted MSCs also promote the regeneration of RGC axons after an optic nerve crush [40]. Interestingly, MSCs can also be effective, indirectly, by inducing the retinal Müller cell production of neurotrophic factors [41]. Overall, the collected data suggest that various subsets of stem cells may be used as a clinical option for the induction of retinal tissue regeneration. Recently, many pilot clinical trials have shown good tolerability and long-term retinal persistence of intravitreally transplanted MSCs [14,42,43].

Recent experimental medical interventions for retinal degeneration are especially focused on the adjustment of extrinsic and intrinsic biological factors which could prevent the progression of vision loss. In animal models of retinal and neuronal degeneration, various growth factors have been tested as possible therapeutic agents, with a particular focus on the use of neurotrophins (NTs) [44,45,46]. This family of proteins participates in the regulation of neuronal cell development, function, and survival. [47]. The cardinal NT components are NGF, BDNF, NT-3, and NT-4. NT deprivation likely contributes to cell death in the retina, and diffusible factors secreted by stem cells have shown protective effects on neurons in animal models of retinal injury [21,29]. Recent studies have highlighted the potential of NT-releasing stem cells in the treatment of several ocular disorders, and the most prominent factor in this venue seems to be BDNF, which, together with other NTs, plays a significant role in the survival of neuronal cells and their downregulation, which is thought to lead to neural degeneration [48]. Additionally, BDNF is important for axonal regeneration, synaptic formation [49], and synaptic plasticity [50]. BDNF binds to the TrkB receptor, which is important for neuron development, survival, and functional maintenance, as well as for neural tissue repair [51]. Until recently, our group has focused on the characterization of subsets of stem cells isolated from different hematopoietic tissues and their ability to secrete NTs that are crucial molecules for neural cell survival, growth, and differentiation [52,53]. We found that different hematopoietic stem cell populations can secrete a broad repertoire of trophic and immunomodulatory cytokines after their transplantation to the neural microenvironment. However, the secreted amounts of NTs vary between different cellular fractions isolated from the human BM or umbilical cord blood [52]. Importantly, in vivo all NTs are short-lived and cannot penetrate easily through the blood-brain barrier and their intravenously or local administration is doubtful to produce long-term therapeutic effects [3]. Therefore, the most efficient method for long-term augmentation of the number of NTs produced by transplanted cells is indispensable. 

In this mode, the transduction of NT genes into MSCs would ensure a sufficient and steady NT expression by these cells. In the current study, we generated genetically modified MSCs for the sustained delivery of BDNF and showed that this approach has the potential to decrease pro-apoptotic signaling in a mouse model of chronic retinal degeneration—rd6 mutant mice. We decided to use that model because of the slow progression of photoreceptor layer loss and because the background of the retinal degeneration is genetic. Furthermore, that model can be used as a mouse model of age-related macular degeneration (AMD). Hence, it seems to be a universal mouse model of retinal neurodegenerative diseases. In cellular therapies, lentiviral vectors are typically used to obtain a stable transgene expression, because of their capacity to transfer exogenous genes into the genome of MSCs [15]. Previously, we employed, with success, the lentiviral vector for the constant transduction of MSC to produce neurotrophin-4 for the preclinical treatment of retinal acute injury [21]. In an experimental protocol, we infected murine MSCs endogenously expressing GFP protein with our self-made Lv-UbC-BDNF lentiviral vector expressing BDNF as a therapeutic gene, with high efficiency (around 95%). Moreover, the morphology and viability of the transduced cells were similar to the uninfected ones. The amount of BDNF protein was significantly higher compared to the uninfected MSCs, indicating an efficient MSC transduction. The in vivo results obtained after three months post-transplantation revealed that BDNF delivered from engineered MSC-BDNF could provide long-lasting biological effects. Transplanted cells successfully migrated towards the injured retinal tissue and were able to incorporate and survive there for at least three months. Moreover, some transplanted MSC-BDNF cells continued to proliferate after homing to the degenerated retina. These results are consistent with previous studies demonstrating that in laser-induced retinal injury, transplanted BM-derived MSCs migrated into the retina where they remained in the proliferative state [54]. We, and other researchers, also observed that after intravitreal injection, the MSC cluster is maintained in the vitreous body for several weeks [40,55], although a small number of MSCs do migrate into the retinal layers, and they are neither tumorigenic nor exhibit uncontrolled growth [36].

According to the above-mentioned findings we hypothesized that genetically engineered MSCs with an overexpression of BDNF that are locally transplanted, may generate an innovative therapeutic strategy for the prolonged preservation of slowly degenerating retinal cells in rd6 mice. The benefits observed in the MSC-BDNF group appear to be a result of the increased retinal cell survival and decreased retinal cell apoptosis. This hypothesis is consistent with previous studies demonstrating BDNF-stimulated survival of differentiated retinal cells resulting from direct BDNF-dependent upregulation of p35 and Bcl-2 anti-apoptotic molecules [56]. The diminished rate of irreversible apoptosis was also confirmed by us at the protein level by detection of Caspase-3, which was undetectable in retinas treated with MSC-BDNF. These data indicate that BDNF may act as an inhibitor of apoptotic cell death in chronic retinal degeneration. Importantly, Junyi et al. showed in a model of optic nerve injury that endogenous BDNF has only a short duration, which is insufficient for the effective prevention of apoptosis in RGCs; therefore, it has no obvious therapeutic effects in optic nerve injury. In contrast, MSC transplantation could provide stable BDNF expression in the infused eyes that could effectively protect against retinal cell apoptosis and cell loss [57]. Likewise, other experimental studies revealed that the transplantation of MSC secreting BDNF has been found to effectively promote retinal cell survival in rodent models of retinal damage [22,23,58,59]. For example, the implantation of BDNF-transduced MSCs into the rat retina was associated with a significant increase in BDNF levels for a period of up to 14 days [60]. Other researchers found that the intravitreal transplantation of BDNF-secreting MSCs in a rat model of chronic ocular hypertension improved retinal cell survival and preserved optic nerve structure [61]. Retinal neuroregeneration may result from the local secretion of neurotrophins by both transplanted engineered MSCs and host cells, with subsequent, even distant, diffusion in the surrounding retinal tissue, thus stimulating different pro-regenerative mechanisms during the process of endogenous neurorepair. Despite many natural barriers in the complex ocular tissue, the experimental results clearly indicate the occurrence of neurotrophins diffusion into the deep retinal layers, where they can exert their biological functions for the homeostasis and the control of neurogenic retinal compartment. The effectiveness of NTs in promoting retinal cell survival depends on the neurotrophic factor receptor (NTR) expression in retinal cells. BDNF activates multiple signaling pathways in neurons via TrkB receptor, and phosphatidylinositol-4,5-bisphosphate 3-kinase (PI3K)/Akt kinase pathways appear crucial in its survival-promoting effect [62]. Interestingly, the expression level of BDNF in the retina appeared to have a direct influence on TrkB mRNA expression levels in our study. We found that the expression of mRNA encoding TrkB was significantly increased in both the MSC-BDNF and MSC alone groups up to three months post-transplantation compared to that in the PBS control group where low expression levels corresponded to low local BDNF production. 

Furthermore, we proved that transplanting MSC-BDNF can influence the retinal function. We found that the local expression of light-sensitive proteins in the photoreceptor layer from the MSC-treated groups was considerably higher than that of the control group, and the b-wave amplitudes of both in rod and cone ERG responses had a better recovery rate in the MSC-BDNF-treated group. The b-wave amplitudes, which reflect the activity of second-order retinal neurons, are a sensitive index of the retinal function as demonstrated in humans and other experimental models [63,64]. The observed improvement in the retinal bioelectrical function indicates that long-term BDNF administration may promote retinal cell survival and substantially decrease the severity of photoreceptor and RPE damage along with the amelioration of functional ERG response in this chronic model of retinal degeneration. Unfortunately, the underlying mechanisms for these positive effects have not yet been elucidated, however the favorable effects of MSC-BDNF transplantation seems to be a result of their ability to control the expression of certain genes, including those involved in signal transduction pathways related to apoptosis. The cellular delivery of BDNF to degenerated retinal tissue had a positive impact in our study, particularly on the expression of the anti-apoptotic proteins Bcl-xL and Mcl-1 that subsequently improved overall neuronal survival. We assume that exogenous BDNF activates a complex signaling cascade that enhances cellular regeneration in slowly degenerating retinas of rd6 mice. This possibility can lead to novel therapeutic opportunities for a wide range of retinal disorders affecting elderly people going blind around the word. However, to meet general quality requirements, cellular components must be at least predefined for the following parameters: Identity, purity, and biological activity. Concerning the complex nature of MSCs, their proper identification and characterization before therapeutic usage must be considered an obligatory constituent of the overall therapeutic strategy [65,66]. Improving, monitoring, and reporting on experimental cell therapies in the field of ophthalmology might help to develop safe and efficient ocular applications for cell-based strategies that can contribute to fully exploiting the widespread potential of cellular therapies. It will also permit avoiding cardinal medical errors that can lead to post-therapeutic iatrogenic blindness, which occurred accidentally in a disastrous clinical treatment of macular degeneration [67]. Therefore, the promise of stem cells as a novel treatment must be tempered by the need to more fully understand stem cell behavior post-transplantation and to assess all the safety risks. This approach strongly emphasizes the importance of selecting the criteria and quality control of the donor cells for cellular and gene combined therapies for ophthalmological disorders, which need to be tested in randomized controlled clinical trials before final legal approval. 

## 4. Materials and Methods

### 4.1. Production of Recombinant Lentivirus Carrying the Human BDNF Gene

The lentiviral vector containing human BDNF (Lv-UbC-BDNF) was constructed using commercially available pFUGW plasmid from Addgene (#14883, Watertown, MA, USA) [68] which enables the expression of a transgene under the human ubiquitin C promoter (UbC). The BDNF coding sequence was obtained from human primary keratinocytes by PCR with specific primers (Forward 5′-CTAGGATCCGCCATGACCATCCTTTTCCTTACTATGG-3′ Reverse 5′-CTAGAATTCAATCCACTATCTTCCCCTTTTAATGG-3′) flanked with BamHI and EcoRI recognition sites. The amplified BDNF sequence was gel-purified, digested, and inserted into the pFUGW plasmid backbone, from which the green fluorescent protein (GFP) sequence was removed with the same pair of restriction endonucleases. The schematic constructions of both plasmids used in this study are presented in Figure 1A and Figure 1B, respectively. The pFUW-BDNF plasmid DNA was identified with restriction endonucleases (EcoRI and BamHI). The correct band for BDNF (765 bp) was confirmed under UV light in agarose gel (Figure 1C). To produce lentiviral vectors, 293T packaging cells were transfected with either pFUW-BDNF or pFUGW (for production of Lv-UbC-GFP vector that served as internal control) as well as both psPAX2 and pMD2.G plasmids using the Transfection Grade Linear Polyethyleneimine (PEI, Polysciences, Inc., Warrington, PA, USA). Vector-containing medium was collected 48 h after cell transfection and filtered through 0.45-µm filters (Merck Millipore, Burlington, MA, USA).

### 4.2. Isolation of MSCs

Bone marrow-derived MSCs were isolated from the femurs and tibias of 4- to 6-week-old transgenic C57BL/6-Tg(CAG-EGFP)1Osb/J mice that express constitutively fluorescent GFP protein, according to the protocol described by Zhu et al. [69] with minor modifications described in Machalińska et al. [21].

### 4.3. Lentiviral Transduction of Murine MSCs

MSCs were genetically engineered to produce and secrete the BDNF neurotrophic factor using a lentiviral vector generated independently as part of the experiment. Briefly, MSCs were plated in 12-well plates (BD Pharmingen, Franklin Lakes, NJ, USA) at a density of 10^5^ cells per well in 500 µL α-MEM (alpha minimal essential medium, Lonza, Bioscience, Walkersville, MD, USA) with 10% FBS and antibiotics and allowed to adhere for 12 h. Then, the α-MEM growth medium was supplemented with 4 µg/well of polybrene (Sigma–Aldrich, Saint Louis, MO, USA). Next, the Lv-UbC-BDNF vector (which was decanted from the 293T-cell culture after transfection) was added to the prepared MCSs for a final volume of 1000 µL. In parallel, a population of control MSCs was engineered with only the Lv-UbC-GFP in the same ratio as the MSC-BDNF to match the viral titer. Viral particles were removed from the MSC cultures after 72 h of exposure by exchanging the culture medium. Next, both engineered MSC populations (MSC-BDNF and MSC-GFP) were maintained as previously described. MSC-GFP cultures were examined for GFP fluorescence at 72 h after initial viral transduction and then passaged after 4 days. Additionally, an enzyme-linked immunosorbent assay (ELISA) was employed from samples taken at several time points during culturing cells to measure the amount of BDNF in the medium collected from MSC-BDNF and MSC cultures.

### 4.4. Flow Cytometry of Murine MSCs

Uninfected MSC and MSC-BDNF were harvested from tissue culture flasks at day 3 post infection and divided into aliquots of 5 × 10^4^ cells. Suspensions were rinsed with sterile PBS, centrifuged at 300 *g* for 8 min, and then rinsed briefly with propidium iodide solution (Sigma-Aldrich) to label dead cells. Next, cells were centrifuged at 300 *g* for 8 min, resuspended in 1-mL PBS, and then analyzed by FACS (LSRII instrument; Becton Dickinson Biosciences, Franklin Lakes, NJ, USA) using CellQuest Software (Becton Dickinson Biosciences). The expression of human MSC markers Sca-1, CD31, CD90 and CD105 was evaluated by flow cytometry in both analyzed cell populations: MSC-BDNF and MSC.

### 4.5. ELISA

Concentration of BDNF in the culture medium from MSC-BDNF cultures was measured using a commercially available high-sensitivity ELISA Quantikine human immunoassay kit for human BDNF (R&D Systems, McKinley Place, MN, USA) according to the manufacturer’s protocol. The absorbance was read at 450 nm using an ELX 808 IU automated Microplate Reader (Bio-Tek Instruments, Inc., Winooski, VT, USA). The results were analyzed using a quadratic log–log curve fit.

### 4.6. Animals and Experimental Procedures

Pathogen-free 5- to 6-month-old mature rd6 mice *n* = 30 (Jackson Laboratory, Bar Harbor, ME, USA) of both sexes and age-matched WT C57BL/6 mice *n* = 10 (Polish Academy of Sciences, Wrocław, Poland) were used in the experiment. Animals were maintained in individually ventilated cages (IVC) in a 12 h light/dark cycle with ad libitum access to food and filtered water. Mice were divided into 2 groups: (1) C57BL/6 (WT) mice that served as control animals, (2) mutant rd6 mice with slow retina degeneration. Among rd6 mice we distinguished mice without transplanted cells (RD6, *n* = 10) and mice with transplanted different types of mesenchymal stromal cells (*n* = 20). Prior to the cell transplantation procedure all mice were anesthetized with an intraperitoneal injection of ketamine (40 mg/kg) and xylazine (4 mg/kg), and their corneas were anesthetized with 0.5% phenylephrine hydrochloride (Alcaine; Alcon, Fort Worth, TX, USA), and pupils were dilated with 1% atropine (Polfa Warszawa, Poland). To the first group of rd6 mice (*n* = 20), 1 µL of PBS containing either cultured 2 × 10^4^ GFP-positive MSCs expressing BDNF (MSC-BDNF) or uninfected GFP-positive MSC cells alone was injected intravitreally into the right and left eye respectively, using a 30 G needle attached to a Hamilton syringe (Hamilton Company, Reno, NV, USA). The second group of rd6 mice and WT mice (*n* = 10) received intravitreal injections of 1 µL of PBS alone into each eye. Murine retinas were monitored for morphological and functional changes by OCT and ERG respectively, for up to 3 months. At 28 days and 3 months after MSC transplantation, 10 of the animals from the first group and 5 from RD6 and WT group were sacrificed for further molecular and histological analysis. All animal procedures were performed according to the European Committee regulations in The Association for Research in Vision and Opthalmology (ARVO) Statement for the Use of Animals in Ophthalmic and Vision Research and were approved by the Local Ethics Committee for matters of animals used for scientific purposes in Szczecin (approval no. 11/2013, approval date: 20 October 2013).

### 4.7. Immunofluorescence Analysis

For cross-sections, the eyes were embedded in paraffin and cut into 5 µm-thick sections. For double immunofluorescence staining, the Tyramide Signal Amplification (TSA, Thermo Fisher Scientific, Waltham, MA, USA) protocol was applied, according to the manufacturer’s protocol. TSA is a useful method that enhances the fluorescence signal and allows the sequential staining with antibodies raised in the same animal species, reducing nonspecific binding. We analyzed the colocalization of the following proteins: BDNF and GFP, PCNA and GFP, opsin blue and rhodopsin, as well as rhodopsin and opsin red/green, according to the scheme of antibodies presented in Table 1. The cross-sections were deparaffinized in xylene (2 × 15 min) followed by hydration in solutions with decreasing ethanol concentrations (100%, 95%, 80%, 70%, and 50%) and antigen retrieval (20 min of boiling in citrate buffer, pH 6.0). For the first set of antibodies, slides were incubated in 3% H_2_O_2_ in PBS for 45 min at room temperature (R_T_) to quench endogenous peroxidase. After blocking in 10% normal serum (goat or donkey, depending on the host species of the secondary antibody) for 30 min at R_T_, the sections were incubated with the first primary antibody in PBS complemented with 1% bovine serum albumin (BSA) at 4 °C overnight. Next, incubation with adequate secondary antibody was performed at R_T_ for 60 min. Fluorescence detection and signal amplification were achieved by staining the sections with Tyramide-Alexa Fluor 488 or Tyramide-Alexa Fluor 594 in amplification buffer/0.0015% H_2_O_2_ at R_T_ for 6 min. For the second set of antibodies, slides were incubated in 3% H_2_O_2_ in PBS for 45 min at room temperature, to quench the horseradish peroxidase activity from the first TSA reaction. After blocking in 10% normal serum (goat or horse, depending on the host species of the secondary antibody) for 30 min at R_T_, the sections were incubated with the second primary antibody in PBS complemented with 1% BSA at 4 °C overnight. Next, incubation with corresponding secondary antibody was performed at R_T_ for 60 min. Finally, the tertiary labeling with Tyramide-Alexa Fluor 488 or Tyramide-Alexa Fluor 594 in amplification buffer/0.0015% H_2_O_2_ was performed at R_T_ for 6 min. Upon termination, sections were counterstained with DAPI solution (Thermo Fisher Scientific, Waltham, MA, USA), mounted, and subjected to microscopy analysis using a LSM700 confocal system (Carl Zeiss, Jena, Germany).

Double immunofluorescence staining using the TSA protocol was used to analyze the colocalization of BDNF and GFP proteins in MSC and MSC-BDNF cultures. Cells grown on the 4-well chamber slides (Thermo Fisher Scientific, Waltham, MA, USA), were fixed in 3.7% paraformaldehyde pH 7.2–7.4 for 20 min, at R_T_, followed by permeabilization with 0.5% Tween 20 in PBS for 10 min, at R_T_. All subsequent staining steps were performed using the antibodies listed in Table 1 and according to the method described.

### 4.8. qRT-PCR

To analyze the mRNA levels for TrkB, BDNF, PCNA and Bcl-xL/BAX ratio, we isolated the total mRNA from WT and rd6 mice retinas at 28 days and 3 months after intravitreal injection of PBS or MSCs using a PARIS Kit (Life Technologies, Paisley, UK). We followed procedures in Machalińska et al. with minor modifications [21]. A 15 µL reaction mixture containing 7.5 µL iQ SYBR Green SuperMix (Bio-Rad), 10 ng of complementary DNA (cDNA) template, and 0.9 µL forward and reverse primers for TRKB, BDNF, BCL-XL, BAX, PCNA and glyceraldehyde 3-phosphate dehydrogenase (GAPDH) were prepared. The oligonucleotide sequences are listed in Table 2. The cDNA was amplified under the following conditions: One cycle at 95 °C for 3 min followed by 40 cycles at 94 °C for 30 s, 61–65 °C for 30 s, and 72 °C for 45 s. Relative quantification of mRNA expression was calculated using comparative Ct method. Relative quantification (ΔΔCt) of mRNA expression was normalized to the reference gene and calculated using the Bio-Rad CFX Manager, Gene Study software (Bio-Rad, Hercules, CA, USA).

### 4.9. Western Blot Analysis

For the Western blot analysis, we followed the method of Machalińska et al. with minor modifications [21]. We used a primary monoclonal immunoglobulin G (IgG) antibodies directed against the BDNF, Caspase-3, phosphoAkt, totalAkt, phosphoMAPK, totalMAPK as follows: Rabbit anti-BDNF monoclonal antibody (at 1:1000 dilution, Abcam, Cambridge, UK, cat no. ab108319), rabbit anti-Caspase-3 monoclonal antibody (at 1:600 dilution, Santa Cruz Biotechnology, CA, USA, cat no. sc-56046), rabbit anti- phospho-Akt monoclonal IgG antibody (at 1:300 dilution, Cell Signaling Technology, Beverly, MA, USA, cat no. 4060S), anti-Akt (pan) monoclonal IgG antibody (at 1:300 dilution, Cell Signaling Technology, Beverly, MA, USA, cat no. 4685S), rabbit anti-phospho-p44/42 MAPK monoclonal IgG antibody (at 1:300 dilution, Cell Signaling Technology, Beverly, MA, USA, cat no. 4370S), rabbit anti-p44/42 MAPK monoclonal IgG antibody (at 1:300 dilution, Cell Signaling Technology, Beverly, MA, USA, cat no. 4695S) and polyclonal anti-GAPDH antibody conjugated with HRP (at a 1:1000 dilution Santa Cruz Biotechnology, Santa Cruz, CA, USA; cat no. sc-20357,). Immunoreactive bands for BDNF and Caspase-3, phosphoAkt, totalAkt, phosphoMAPK and totalMAPK were detected using a horseradish peroxidase-conjugated goat anti-rabbit secondary antibody (goat F(ab) anti-rabbit IgG H&L (HRP), Abcam, Cambridge, UK, cat no. ab7171, dilution 1:1000). Chemiluminescence detection was performed using Western Bright Sirius Western Blotting HRP Substrate (Advansta, CA, USA).

### 4.10. Multiplex Immunoassay

Mcl-1/Bak dimer and Bcl-xl/Bak dimer concentrations were quantified in homogenates of collected retinas by multiplex fluorescent bead-based immunoassays (Luminex Corporation, Austin, TX, USA) using commercial Bio-Plex Pro™ RBM Apoptosis Multiplex Assays, Panel 3 Analytes (Bio-Rad, Hercules, CA, USA) following the manufacturer’s instructions. To standardize the final concentration-values, we confined the measurement of the total protein to each sample.

### 4.11. Electroretinography

Scotopic and photopic ERGs were recorded from WT mice, rd6 mice, rd6 mice after MSC transplantation, and rd6 mice after MSC-BDNF transplantation at 28 days and 3 months after injection. We followed the method of Machalińska et al. [21].

### 4.12. Spectral-Domain Optical Coherence Tomography (SD-OCT) Imaging

SD-OCT scans were performed on mice at 28 days and 3 months after injection as described before [21]. The reference arm was placed at approximately 1197 mm. In each eye 4 images were taken of specific regions, using the optic nerve as a landmark. Rectangular scans (1.5 mm × 1.5 mm, 1280 a-scans/b-scan × 150 b/scans) were obtained.

### 4.13. Statistical Methods

The significance of differences between experimental groups was assessed using the Kruskal–Wallis test followed by the Mann–Whitney test. *p* < 0.05 was considered statistically significant. Data are presented as the mean ± SD. Student’s *T*-test was used to assess the changes between groups in ELISA results.

## 5. Conclusions

In summary, our findings suggest that MSCs genetically modified to produce BDNF neurotrophin are a good candidate for a potential therapeutic strategy to protect degenerating retina. The results of this study also shed light on mechanisms of chronic retinal degeneration, identifying exogenous BDNF as a prospective regulatory factor for retinal tissue. However, it is crucial to assess the long-term safety and efficacy of the current genetically modified MSC-based cell transplantation approach in humans. Our results thus give rise to a potential successful clinical application of genetically modified MSC in elderly patients, which may be able to improve the outcomes in chronic retinal degenerative diseases of humans in the immediate future.

## Figures and Tables

**Table 1:** List of antibodies employed in double immunofluorescence staining technique.

Coexpression	Method	First Set		Second Set	
		Antibody	Dilution	Antibody	Dilution
	I Ab *	rabbit anti-BDNF ^1^	1:100	rabbit anti-GFP ^1^	1:100
BDNF and GFP	II Ab **	goat anti-rabbit HRP ^2^	1:100	goat anti-rabbit HRP ^2^	1:100
	Detection	Tyramide Alexa-Fluor 594 ^2^	1:100	Tyramide Alexa-Fluor 488 ^3^	1:100
	I Ab	goat anti-PCNA ^4^	1:100	rabbit anti-GFP ^1^	1:100
PCNA and GFP	II Ab	donkey anti-goat HRP ^5^	1:100	chicken anti-rabbit HRP ^6^	1:100
	Detection	Tyramide Alexa-Fluor 594 ^2^	1:100	Tyramide Alexa-Fluor 488 ^3^	1:100
	I Ab	rabbit anti-opsin blue ^5^	1:100	mouse anti-rhodopsin ^1^	1:1000
opsin blue and rhodopsin	II Ab	goat anti-rabbit HRP ^2^	1:100	goat anti-mouse HRP ^3^	1:100
	Detection	Tyramide Alexa-Fluor 594 ^2^	1:100	Tyramide Alexa-Fluor 488 ^3^	1:100
	I Ab	mouse anti-rhodopsin ^1^	1:1000	rabbit anti-opsin red/green ^5^	1:250
rhodopsin and opsin red/green	II Ab	goat anti-mouse HRP ^3^	1:100	goat anti-rabbit HRP ^2^	1:100
	Detection	Tyramide Alexa-Fluor 488 ^3^	1:100	Tyramide Alexa-Fluor 594 ^2^	1:100

Manufacturers: ^1^ Abcam (Cambridge, UK); ^2^ Thermo Fisher Scientific (Waltham, MA, USA), part of TSA Kit #15; ^3^ Thermo Fisher Scientific (Waltham, MA, USA), part of TSA Kit #2; ^4^ Santa Cruz Biotechnology (Dallas, TE, USA); ^5^ EMD Millipore (Billerica, MA, USA); ^6^ Thermo Fisher Scientific (Waltham, MA, USA). * I Ab- primary antibody, ** II Ab- secondary antibody.

**Table 2:** List of oligonucleotide sequences used in the experiment.

Gene	Primer	Sequence
mGAPDH	Forward	5′-AGGTCGGTGTGAACGGATTT-3′
	Reverse	5′-TGTAGACCATGTAGTTGAGGT-3′
mBDNF	Forward	5′-GCACTGGAACTCGCAATGC-3′
	Reverse	5′-GTAAGGGCCCGAACATACGA-3′
mTRKB	Forward	5′-TTGACCCGGAGAACATCACG-3′
	Reverse	5′-CCACAAACTTTAAGCCGGAATCC-3′
mPCNA	Forward	5′-CTTGGTACAGCTTACTCTGCG-3′
	Reverse	5′-AGTTGCTCCACATCTAAGTCCAT-3′
mBCL-XL	Forward	5′-GACAAGGAGATGCAGGTATTGG-3′
	Reverse	5′-TCCCGTAGAGATCCACAAAAGT-3′
mBAX	Forward	5′-TGAAGACAGGGGCCTTTTTG-3′
	Reverse	5′-AATTCGCCGGAGACACTCG-3′

## Abbreviations

qRT-PCR	Quantitative Reverse Transcription Polymerase Chain Reaction
GAPDH	Glyceraldehyde 3-phosphate dehydrogenase
Casp-3	Caspase-3
Bcl-xL	B-cell lymphoma-extra-large protein
Bcl-2	B-cell lymphoma 2 protein
Mcl-1	Induced myeloid leukemia cell differentiation protein
BDNF	Brain-derived Neurotrophic Factor
MFRP	Membrane-type Frizzled Related Protein
TrkA	Tyrosine Receptor Kinase A
TrkB	Tyrosine Receptor Kinase B
TrkC	Tyrosine Receptor Kinase C
PCNA	Proliferating Cell Nuclear Antigene
DAPI	4′,6-diamidino-2-phenylindole
Sca-1	Stem cell antigen 1
PI3K	Phosphatidylinositol-4,5-bisphosphate 3-kinase
MSCs	Mesenchymal Stromal Cells
Bax	Bcl-2 associated X protein
Bak	Bcl-2 homologous antagonist killer
Akt	Protein kinase B
ERG	Electroretinography
GFP	Green Fluorescent Protein
PBS	Phosphate Buffered Saline
rd6	Retinal degeneration 6
OCT	Optical Coherence Tomography
RPE	Retinal Pigment Epithelium
RGC	Retinal Ganglion Cells
NGF	Neural Growth Factor
CNS	Central Nervous System
ALS	Amyotrophic Lateral Sclerosis
NT-3	Neurotrophin-3
NT-4/5	Neurotrophin-4/5
NTs	Neurotrophins
BM	Bone-Marrow
R_T_	Room Temperature
SD	Standard Deviation
